# Supplementary material for: Pleiotropic Effects of the Protease-Activated Receptor 1 (PAR1) Inhibitor, Vorapaxar, on Atherosclerosis and Vascular Inflammation
Source: Cells. 2021 Dec 13;10(12):3517. doi: 10.3390/cells10123517 (PMC8700178; doi:10.3390/cells10123517)
Supplement: Supplementary file 1 [file cells-10-03517-s001.zip › cells-1498154 - suppl. figures.pdf]

Supplemental Figures

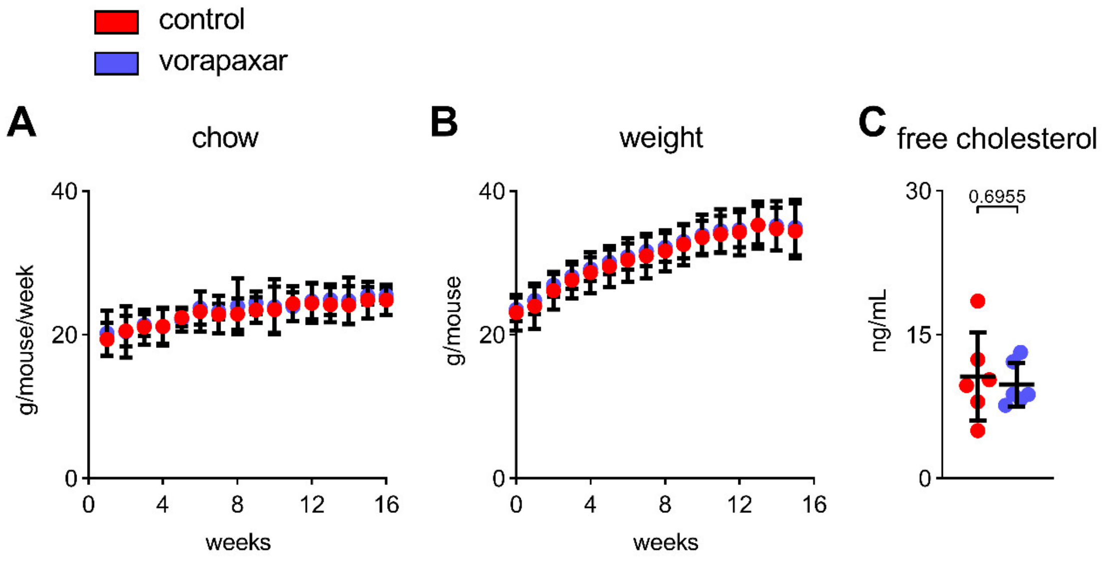

Figure S1. Vorapaxar does not alter food intake, weight gain, or circulating cholesterol. Monitoring of (**A**) food intake (g/mouse/week) and (**B**) weight gain (g/mouse). (**C**) Plasma concentration of free cholesterol after 16 weeks on diet, *n* = 6. Results are expressed as mean with SD (**A**,**B**) or as single values (*n* = 6), mean with SD (**C**).

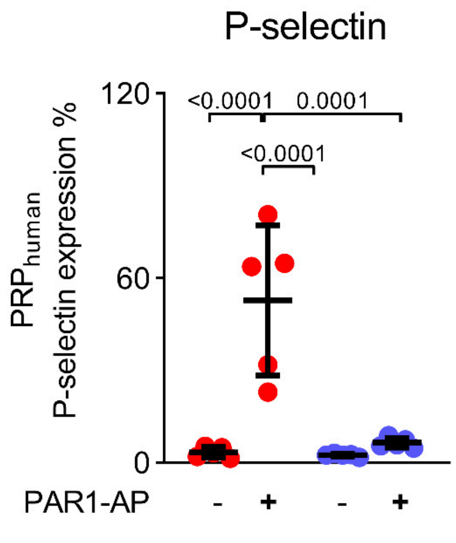

Figure S2. Vorapaxar maintains its PAR1-inhibiting capacity in blood after oral uptake in mice. Pooled human platelets (PRP) were resuspended in isolated plasma (PPP) from mice that received WD ± vorapaxar (*n* = 5) for 7 days and stimulated with the PAR1-activating peptide (PAR1-AP). The expression of P-selectin in platelets was measured by flow cytometry.

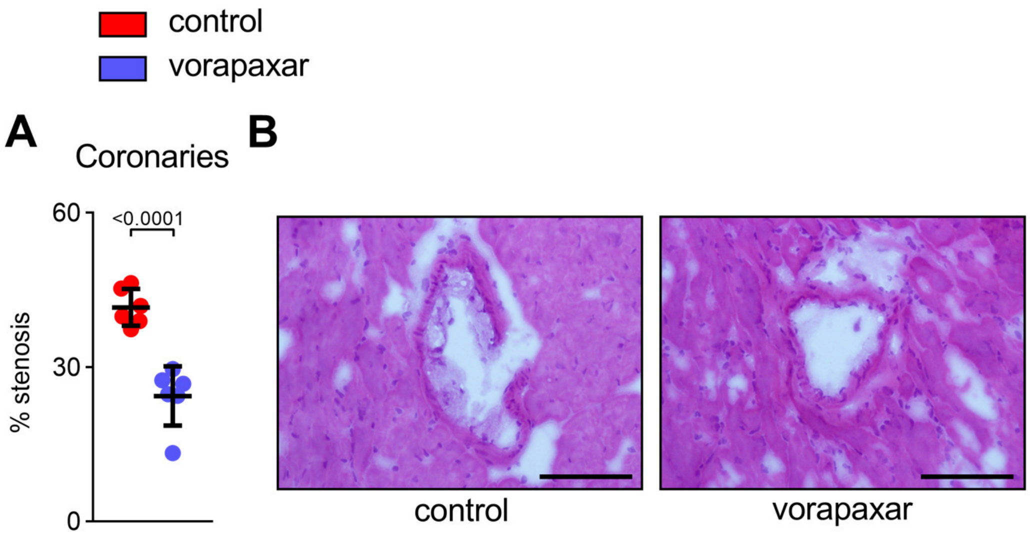

Figure S3. Vorapaxar reduces de-novo atherosclerosis in ApoEko mice fed an atherogenic diet. (**A**) Coronary artery stenosis (% lumen reduction). Representative images, scale bars 80 µm (**B**). Results are expressed as single values (n = 6), mean with SD.

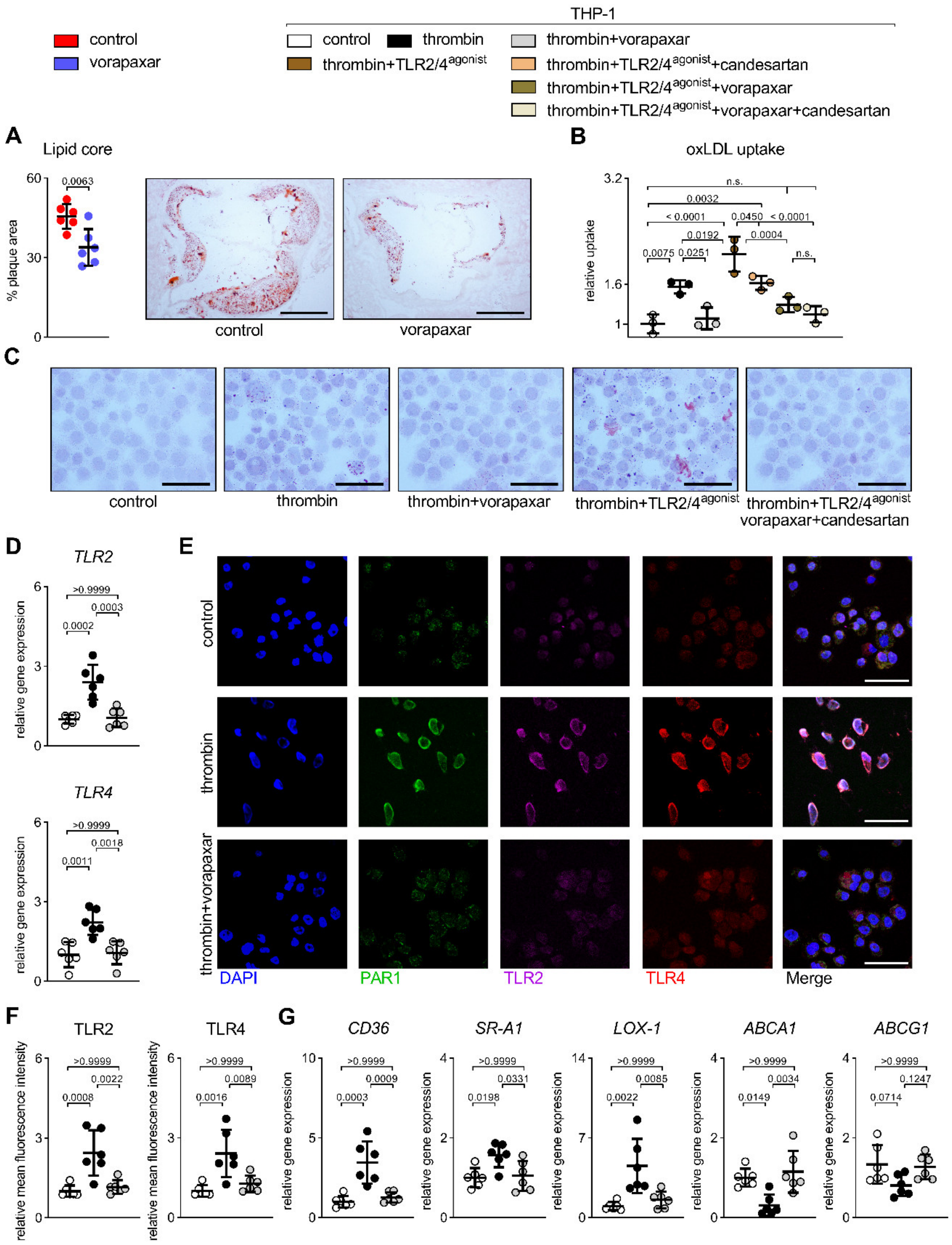

Figure S4. PAR1 inhibition with vorapaxar impairs foam-cell formation. (**A**) Treatment with vorapaxar is associated with reduction in plaque lipid content. The % Oil red O-positive area within aortic sinus of ApoEko mice. (**B**,**C**) Activation of PAR1, TLR2, and TLR4-activation increases oxLDL uptake (% Oil red O-positive intracellular area relative to control) in THP-1 cells. Cells were pretreated with thrombin, thrombin + vorapaxar, thrombin + TLR2/4-agonists, or a combination of thrombin + TLR2/4-agonists + vorapaxar + candesartan (combined TLR2/4-antagonist). (**D**) Effect of PAR1 activation on transcription of *TLR2* and *TLR4* in THP-1 cells. (**E**) Increased surface expression and colocalization of PAR1 with TLR2 and TLR4 under thrombin stimulation. (**F**) Effect of PAR1 activation on expression (relative mean fluorescence intensity) of TLR2 and TLR4 in THP-1 cells. (**G**) Thrombin-stimulated PAR1 is involved in

regulation of receptors that are involved in cellular lipid metabolism (*CD36*, *SR-A1*, *LOX-1*, *ABCA1*, *ABCG1*). Representative images, scale bars 400 µm (**A**), 50 µm (**C**,**E**). Results are expressed as single values (mice $n = 6$, THP-1 cells $n = 3–6$), mean with SD. n.s. = not significant.
