# Supplementary material for: Pleiotropic Effects of the Protease-Activated Receptor 1 (PAR1) Inhibitor, Vorapaxar, on Atherosclerosis and Vascular Inflammation
Source: Cells. 2021 Dec 13;10(12):3517. doi: 10.3390/cells10123517 (PMC8700178; doi:10.3390/cells10123517)
Supplement: Supplementary file 1 [file cells-10-03517-s001.zip › cells-1498154 - suppl. materials.pdf]

Supplemental Material

Animals (in vivo studies)

Species	Vendor or Source	Background Strain	Sex
mice: Apoe^{tm1Unc}	Charles River Laboratories, Wilmington, MA, USA	C57BL/6	male

Antibodies

Target antigen	Vendor or Source	Catalog #	Working concentration
CD3	Santa Cruz, Dallas, TX, USA	sc-1127	1:100
CD11b+/Mac-1+	ImmunoTools, Friesoythe, Germany	21819111	1:500
CD68	Abcam, Cambridge, UK	ab53444	1:75
CD80	Becton, Dickinson and Company, Franklin Lakes, NJ, USA	553766	1:50
CD206	Abcam, Cambridge, UK	ab64693	1:100
PAR1	R&D Systems, Minneapolis, MN, USA	MAB3855	1:50
PAR1 (Cleaved-Ser42), N-Terminal	Sigma-Aldrich, St. Louis, MO, USA	SAB4500823	1:400
Secondary Antibody, Alexa Fluor 405	Thermo Fisher Scientific, Waltham, MA, USA	Catalog # A-31556	1:500
P-selectin	Santa Cruz, Dallas, TX, USA	SC8419	1:100
TLR2	Novus Biologicals, Littleton, CO, USA	IMG-410A	1:100
TLR4	R&D Systems, Minneapolis, MN, USA	AF1478	1:125
TLR4	Novus Biologicals, Littleton, CO, USA	IMG578A	1:200
VCAM-1	Becton, Dickinson and Company, Franklin Lakes, NJ, USA	550547	1:100

DNA/cDNA Clones

Clone Name	Sequence	Source / Repository
ABCA1	Hs01059137_m1	Thermo Fisher Scientific, Waltham, MA, USA
ABCG1	Hs00245154_m1	
CCL2	Mm00441242_m1	
CD36	Hs00354519_m1	
E-selectin	Mm00441278_m1	
18s	Mm03928990_g1	
18s	Hs99999901_s1	
IFN-γ	Mm00801778_m1	
IL-1β	Mm00434228_m1	
IL-12B	Mm99999067_m1	
IL2	Mm00434256_m1	
LOX-1	Hs01552593_m1	
SR-A1	Hs00398296_g1	
TF	Hs00175225_m1	

TF	Mm00438855_m1	
TLR2	Hs02621280_s1	
TLR4	Hs00152939_m1	
TLR2	Mm00442346_m1	
TLR4	Mm00445273_m1	
TNF-α	Mm00443258_m1	
VCAM-1	Hs01003372_m1	
VCAM-1	Mm0049197_m1	

Cultured Cells

Name	Vendor or Source
THP-1	ATCC # TIB 202, Sigma-Aldrich, St. Louis, MO, USA

Other

Description	Source / Repository
Candesartan	Tocris, Bristol, UK<br>Cat. No. 4791
Cholesterol	Elabscience, Elabscience<br>E-EL-M2608
C-Reactive Protein/CRP DuoSet ELISA	R&D, Minneapolis, MN, USA<br>Catalog # DY1707
E-Selectin/CD62E DuoSet ELISA	R&D, Minneapolis, MN, USA<br>Catalog # DY724
ICAM-1/CD54 DuoSet ELISA	R&D, Minneapolis, MN, USA<br>Catalog # DY720
IL-6 DuoSet ELISA	R&D, Minneapolis, MN, USA<br>Catalog # DY206
Isoflurane	AbbVie, Lake Bluff, IL, USA
Oil red O Staining kit	Abcam, Cambridge, UK
PECAM-1/CD31 Dynabeads™	Invitrogen, Waltham, MA, USA
LPS	Sigma-Aldrich, St. Louis, MO, USA<br>#L2630
oxLDL	Bio-Rad, Hercules, CA, USA<br>5685-3557
Pam3CSK4	R&D, Minneapolis, MN, USA<br>Catalog 4633/1
PF4/CXCL4 ELISA	R&D, Minneapolis, MN, USA<br>Catalog # MCX400
P-selectin ELISA	R&D, Minneapolis, MN, USA<br>Catalog # MPS00
TAT ELISA	Novus Biologicals, Littleton, CO, USA<br>NBP2-75962

TFLLR-NH2	Tocris, Bristol, UK<br>1464
Thrombin	Sigma-Aldrich, St. Louis, MO, USA<br>T9326
TNF-alpha Quantikine HS ELISA	R&D, Minneapolis, MN, USA<br>Catalog # HSTA00E
Western type diet	ssniff, Soest, Germany<br>no. E15721-34
VCAM-1/CD106 DuoSet ELISA	R&D, Minneapolis, MN, USA<br>Catalog # DY809
Vorapaxar	Selleckchem, Houston, TX, USA
